# Supplementary material for: Reconciling scaling of the optical conductivity of cuprate superconductors with Planckian resistivity and specific heat
Source: Nat Commun. 2023 May 26;14:3033. doi: 10.1038/s41467-023-38762-5 (PMC10220041; doi:10.1038/s41467-023-38762-5)
Supplement: Supplementary file 1 — Supplementary information [file 41467_2023_38762_MOESM1_ESM.pdf]

Supplementary Information

Reconciling Scaling of the Optical Conductivity of Cuprate Superconductors with Planckian Resistivity and Specific Heat

Bastien Michon,[1,2,3] Christophe Berthod,[1] Carl Willem Rischau,[1] Amirreza Ataei,[4] Lu Chen,[4] Seiki Komiya,[5] Shimpei Ono,[5] Louis Taillefer,[4,6] Dirk van der Marel [1,†], and Antoine Georges [1,7,8,9,†]

[1]*Department of Quantum Matter Physics, University of Geneva, 24 quai Ernest-Ansermet, 1211 Geneva, Switzerland*
[2]*Department of Physics, City University of Hong Kong, 83 Tat Chee Avenue, Kowloon, Hong Kong, China*
[3]*Hong Kong Institute for Advanced Study, City University of Hong Kong, 83 Tat Chee Avenue, Kowloon, Hong Kong, China*
[4]*Institut Quantique, Département de Physique & RQMP, Université de Sherbrooke, Sherbrooke, Québec, Canada*
[5]*Energy Transformation Research Laboratory, Central Research Institute of Electric Power Industry, 2-6-1 Nagasaka, Yokosuka, Kanagawa, Japan*
[6]*Canadian Institute for Advanced Research, Toronto, Ontario, Canada*
[7]*Collège de France, 11 place Marcelin Berthelot, 75005 Paris, France*
[8]*Center for Computational Quantum Physics, Flatiron Institute, New York, New York 10010, USA*
[9]*CPHT, CNRS, École Polytechnique, IP Paris, F-91128 Palaiseau, France*
[†]*Corresponding Author: dirk.vandermarel@unige.ch*
[†]*Corresponding Author: antoine.georges@college-de-france.fr*
(Dated: May 5, 2023)

A. HIGHER ENERGY TRANSITIONS AND THE VALUE OF ϵ_∞

The theoretical scaling *Ansatz* that we have used to interpret the optical conductivity data only applies at low energy. Obviously, there are also higher-energy transitions that are not described by this *Ansatz*. In this section, we show that these higher-energy transitions yield a contribution to ϵ_∞ which is of order unity. This observation is helpful in clarifying why the value of ϵ_∞ that provides the best possible scaling of the data is smaller than both the one deduced by analyzing the integrated spectral weight and the value more commonly admitted for this class of materials. We use a simple model of these high-energy transitions involving upper and lower Hubbard bands.

We consider electrons characterized by a spectral function of the form $A(\mathbf{k},\varepsilon) = A_Z(\xi_\mathbf{k},\varepsilon) + A_\Delta(\varepsilon)$. The term $A_Z(\xi_\mathbf{k},\varepsilon)$ represents low-energy quasiparticles with a band dispersion $\xi_\mathbf{k}$ (measured from the chemical potential) and a momentum-independent self-energy. The sum rule for this term is $\int_{-\infty}^{\infty} d\varepsilon A_Z(\xi_\mathbf{k},\varepsilon) = Z < 1$, where Z is the quasiparticle residue. The remaining spectral weight $1-Z$ is assumed to reside in lower and upper Hubbard-like bands, the former being fully occupied around energy $-\Delta_1$ and the latter being empty around energy $+\Delta_2$. For simplicity, we describes these bands as dispersion-less and write the corresponding spectral function as $A_\Delta(\varepsilon) = p_1\delta(\varepsilon+\Delta_1)+p_2\delta(\varepsilon-\Delta_2)$ with Δ_1 and Δ_2 positive. In a one-band model, the condition $p_1 + p_2 = 1 - Z$ must be obeyed to ensure the total sum rule $\int_{-\infty}^{\infty} d\varepsilon A(\xi_\mathbf{k},\varepsilon) = 1$. In the cuprates, the hybridization of O $2p$ and Cu $3d$ electrons implies that the total spectral weight of the Hubbard bands is generally larger than $1-Z$, but difficult to estimate precisely. We will therefore keep p_1 and p_2 as independent parameters in the following.

The optical conductivity is split into a low-frequency contribution $\sigma_\mathrm{L}(\omega)$ determined by the transitions within the quasiparticle band and a high-frequency contribution $\sigma_\mathrm{H}(\omega)$ containing the transitions between the Hubbard bands and the quasiparticle band, as well as the transitions between the Hubbard bands. The dielectric function is $\epsilon(\omega) = 1 - \sigma(\omega)/(i\epsilon_0\omega)$. The 1 represents the dielectric response of the vacuum with vanishing conductivity. The quantity $\epsilon_\mathrm{L}(\omega) = 1 - \sigma_\mathrm{L}(\omega)/(i\epsilon_0\omega)$ therefore represents the dielectric function of a vacuum dressed by the low-energy quasiparticles, without the polarizability associated with the high-frequency transitions, which is captured by $\epsilon_\mathrm{H}(\omega) = -\sigma_\mathrm{H}(\omega)/(i\epsilon_0\omega)$. If the low- and high-frequency transitions are well separated—we will assume they are—the function $\epsilon_\mathrm{H}(\omega)$ approaches a real constant in the low-frequency region, where the intra-band quasiparticle transitions take place, and this term can be replaced by its zero-frequency value, e.g., $\epsilon_\mathrm{H}(\omega) \approx \epsilon_\mathrm{H}(0) = \Delta\epsilon_\infty$ with ∞ recalling that this contribution originates from high-frequency transitions. The corresponding approximate dielectric function is commonly written as $\epsilon(\omega) \approx \epsilon_\infty - \sigma_\mathrm{L}(\omega)/(i\epsilon_0\omega)$ with $\epsilon_\infty = 1 + \Delta\epsilon_\infty$. Our goal is to calculate $\Delta\epsilon_\infty$, which is formally defined as

$$\Delta\epsilon_\infty = \mathrm{Re}\,\epsilon_\mathrm{H}(0) = -\frac{1}{\epsilon_0}\lim_{\omega\to0}\frac{\mathrm{Im}\,\sigma_\mathrm{H}(\omega)}{\omega} = -\frac{1}{\epsilon_0}\lim_{\omega\to0}\frac{d}{d\omega}\mathrm{Im}\,\sigma_\mathrm{H}(\omega). \quad \text{(S1)}$$

Using the Kramers–Kronig relation and the fact that $\mathrm{Re}\,\sigma_\mathrm{H}(\omega)$ is an even function of ω, this may also be expressed as

$$\Delta\epsilon_\infty = \frac{2}{\pi\epsilon_0}\int_0^\infty d\omega\,\frac{\mathrm{Re}\,\sigma_\mathrm{H}(\omega)}{\omega^2}. \quad \text{(S2)}$$

This expression only makes sense if $\sigma_{\mathrm{H}}(\omega)$ vanishes sufficiently fast at $\omega=0$ or is gapped. This condition may be considered as a necessary one for the separation into low- and high-frequency degrees of freedom to be meaningful.

The optical conductivity is related to the electron spectral function and transport function $\Phi(\xi)$ via

$$\sigma(\omega)=\frac{i}{\omega}\int_{-\infty}^{\infty}d\xi\,\Phi(\xi)\int_{-\infty}^{\infty}d\varepsilon_1 d\varepsilon_2 A(\xi,\varepsilon_1)A(\xi,\varepsilon_2)\times\frac{f(\varepsilon_1)-f(\varepsilon_2)}{\hbar\omega+\varepsilon_1-\varepsilon_2+i0},\quad (S3)$$

which reduces to Eq. (10) of the main text if $\Phi(\xi)$ is replaced by $\Phi(0)$ [1]. As we are not interested here in the temperature dependence of $\Delta\epsilon_\infty$, we set $T=0$ and deduce

$$\mathrm{Re}\,\sigma(\omega)=\frac{\pi}{\omega}\int_{-\infty}^{\infty}d\xi\,\Phi(\xi)\int_{-\hbar\omega}^{0}d\varepsilon A(\xi,\varepsilon)A(\xi,\varepsilon+\hbar\omega). \quad (S4)$$

There are three contributions to $\mathrm{Re}\,\sigma_{\mathrm{H}}(\omega)$: one describes the transitions from the lower Hubbard band to the quasiparticle band $[A(\xi,\varepsilon)=p_1\delta(\varepsilon+\Delta_1), A(\xi,\varepsilon+\hbar\omega)=A_Z(\xi,\varepsilon+\hbar\omega)]$; one describes the transitions from the quasiparticle band to the upper Hubbard band $[A(\xi,\varepsilon)=A_Z(\xi,\varepsilon), A(\xi,\varepsilon+\hbar\omega)=p_2\delta(\varepsilon+\hbar\omega-\Delta_2)]$; one describes the transitions from the lower to the upper Hubbard bands $[A(\xi,\varepsilon)=p_1\delta(\varepsilon+\Delta_1), A(\xi,\varepsilon+\hbar\omega)=p_2\delta(\varepsilon+\hbar\omega-\Delta_2)]$. The three other processes (quasiparticle to lower Hubbard, higher Hubbard to quasiparticle, and higher Hubbard to lower Hubbard) are suppressed at $T=0$. The real part of the high-frequency conductivity is, therefore,

$$\mathrm{Re}\,\sigma_{\mathrm{H}}(\omega)=\frac{\pi}{\omega}\int_{-\infty}^{\infty}d\xi\,\Phi(\xi)\Big[p_1\theta(\hbar\omega-\Delta_1)A_Z(\xi,-\Delta_1+\hbar\omega)+p_2\theta(\hbar\omega-\Delta_2)A_Z(\xi,\Delta_2-\hbar\omega)+p_1p_2\delta(\hbar\omega-\Delta_1-\Delta_2)\Big],\quad (S5)$$

where $\theta(\varepsilon)$ is the Heaviside step function. $\Delta\epsilon_\infty$ follows from Eq. (S2) as

$$\Delta\epsilon_\infty=\frac{2\hbar^2}{\epsilon_0}\int_{-\infty}^{\infty}d\xi\,\Phi(\xi)\left[p_1\int_0^{\infty}d\varepsilon\frac{A_Z(\xi,\varepsilon)}{(\varepsilon+\Delta_1)^3}+p_2\int_{-\infty}^{0}d\varepsilon\frac{A_Z(\xi,\varepsilon)}{(\Delta_2-\varepsilon)^3}+\frac{p_1p_2}{(\Delta_1+\Delta_2)^3}\right].\quad (S6)$$

Since $A_Z(\xi,\varepsilon)$ is peaked near $\xi=\varepsilon$ while $\Phi(\xi)$ is a slow function of energy, we have $\int_{-\infty}^{\infty}d\xi\,\Phi(\xi)A_Z(\xi,\varepsilon)\approx\Phi(\varepsilon)\int_{-\infty}^{\infty}d\xi A_Z(\xi,\varepsilon)=\Phi(\varepsilon)Z$. The remaining ε integral is cut at the non-interacting bandwidth D, beyond which $\Phi(\varepsilon)$ vanishes. For an order-of-magnitude estimate, we take $\Phi(\varepsilon)=\Phi(0)$ for $|\varepsilon|<D$ and zero otherwise, which leads to

$$\Delta\epsilon_\infty\sim\frac{\hbar^2\Phi(0)}{\epsilon_0\Delta_1^2}\left[Zp_1\frac{D(D+2\Delta_1)}{(D+\Delta_1)^2}+Zp_2\frac{\Delta_1^2}{\Delta_2^2}\frac{D(D+2\Delta_2)}{(D+\Delta_2)^2}+4p_1p_2\frac{D}{\Delta_1(1+\Delta_2/\Delta_1)^3}\right].\quad (S7)$$

Using our model parameter $\Phi(0)=1.33\times10^7\epsilon_0\mathrm{THz}^2$, $\Delta_1=1.46$ eV, which corresponds to the peak in Fig. 1**a** of the main text, $D=4t=1.2$ eV, and $\Delta_2\gg\Delta_1,D$, we arrive at $\Delta\epsilon_\infty\sim1.89Zp_1$. Hence, we see that the transitions involving the Hubbard bands yield a contribution to ϵ_∞ that is of order unity. A more quantitative assessment is beyond the applicability of this simple model and would also require a quantitative determination of the weights Z and p_1.

B. FREQUENCY-TEMPERATURE SCALING OF THE OPTICAL DATA

Here, we show that the frequency and temperature dependencies of the optical scattering rate and optical mass enhancement indicate a Planckian dissipation (with single-particle self-energy exponent $\nu=1$) — despite the fact that the modulus of the optical conductivity displays an exponent $\nu^*<1$ as seen in Fig. 3 of the main text. Based on the local models considered in the present work and discussed in detail in Supplementary Information Sec. C and D, we expect that the scattering rate and mass enhancement scale according to $1/\tau(\omega)\approx T^\nu f_\tau(\omega/T)$ and $m^*(\omega)-m^*(0)\approx T^{\nu-1}f_m(\omega/T)$. If not for the approximate signs and the shift by $m^*(0)$, these scaling laws would imply that the conductivity behaves ideally as $1/\sigma(\omega)=T^\nu F(\omega/T)$ with $F(x)=f_\tau(x)-ixf_m(x)$ [see Eq. (15) of the main text]. The numerical simulations show that this property is not obeyed by Eqs. (10), (7), and (S18), while the approximate scaling laws with the mass shift by $m^*(0)$ are numerically well obeyed (see Fig. 5 of the main text and Supplementary Fig. 9). The subtraction of $m^*(0)$ in order to observe ω/T scaling is mandatory for $\nu=1$, because $m^*(0)$ varies as $\ln T$ in that case, while for $\nu<1$ it is optional, because $m^*(0)\sim T^{\nu-1}$.

The measured infrared dielectric function of LSCO at doping $p=0.24$ is displayed in Supplementary Fig. 1. We emphasize in the main text that the frequency dependence of $1/\tau$ and m^*/m extracted from the dielectric function depends on the value chosen for the background dielectric constant. Here, in order to test the presence of scaling laws in the data, we optimize the value of ϵ_∞ to achieve the best scaling collapse. The results of this procedure for $1/\tau$ are displayed in Supplementary Fig. 2**a**. For each value of ν, we minimize a cost function representing the quality of the collapse and we deduce the optimal ϵ_∞ indicated in each panel. We use the optical data in the range $T>30$ K and $\hbar\omega<0.4$ eV for this analysis. The data for $T=30$ K and below are strongly affected by the loss of information due to the sharpening of the Drude peak below our observation

limit of 2.5 meV and the opening of the superconducting gap below $T_c = 19$ K. Furthermore, interband transitions come into play above 0.4 eV. The figure shows that the collapse improves upon increasing ν towards $\nu = 1$. For $\nu \lesssim 0.8$, the optimal ϵ_∞ is larger than the largest measured infrared dielectric function (Supplementary Fig. 1) and the collapse is poor. Supplementary Fig. 2**b** illustrates how the scaling collapse for $\nu = 1$ depends on the value of ϵ_∞.

In order to test the scaling laws for m^*, we must determine $m^*(0)$. The low-frequency noise prevents us from extracting this information directly from the data. We therefore treat $m^*(0)$ for each temperature as a variable that we adjust, like ϵ_∞, for optimal collapse. The results are displayed in Supplementary Fig. 3. Supplementary Fig. 3**a** shows a systematic improvement of the collapse with ν approaching unity. All temperatures were included in this analysis, which allows us to get the full T-dependence of $m^*(0)$, as shown in Supplementary Fig. 3**b**. Note that this procedure does not set an absolute scale for $m^*(0)$, unless the scale of m^* is fixed by a choice of the spectral weight K. For $\nu = 1$, the optimal $m^*(0)$ values align on a straight line, indicating a precise $\ln T$ behavior consistent with the behavior of the quasiparticle mass revealed by the electronic specific heat.

The optimizations presented so far establish that the self-energy exponent takes the value $\nu = 1$. However, the optimal ϵ_∞ determined from $1/\tau$ and m^* differ. One sees in Supplementary Fig. 2**b** that the collapse of $1/\tau$ quickly deteriorates as ϵ_∞ increases beyond 3 and would be badly broken for $\epsilon_\infty = 6.66$, as required for optimal collapse of the mass. In fact, the large values of ϵ_∞ stem from the m^* data above ~ 0.2 eV. It is seen in Fig. 1**d** of the main text that a change of behavior occurs around 0.22 eV in the mass enhancement data. The data above 0.22 eV scale in a different way, as also seen in Fig. 2**d** of the main text. We find that the optimal ϵ_∞ drops to a value close to 3 if those data are ignored. In contrast, the optimal ϵ_∞ for $1/\tau$ does not change appreciably upon varying the data range below 0.4 eV. We therefore perform a common optimization for both $1/\tau$ and m^* by minimizing the sum of the two cost functions (normalized to the value at their respective minimum), considering energies up to 0.4 eV and temperatures larger than 30 K for $1/\tau$, while keeping all temperatures but only energies up to 0.22 eV for m^*. The resulting optimum is $\epsilon_\infty = 2.76$, very close to the optimum for $1/\tau$, and the resulting zero-frequency masses and scaling collapses are displayed in Fig. 2 of the main text. Note that these technical choices are inessential: by keeping data up to 0.22 eV or 0.4 eV for both $1/\tau$ and m^*, we obtain very similar optimal ϵ_∞ of 2.91 and 3.03, respectively, and virtually identical values of $m^*(0)$.

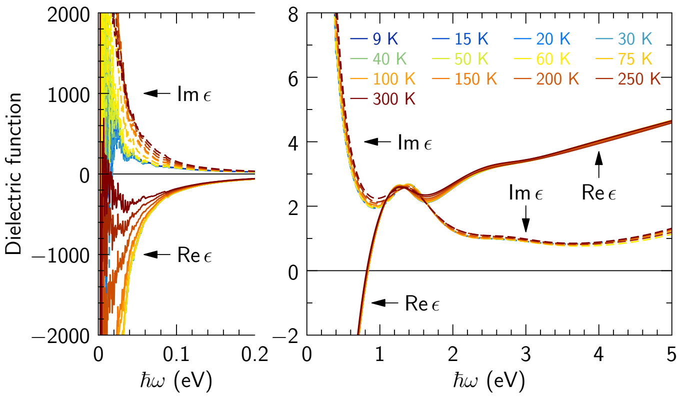

Supplementary Fig. 1. Complex dielectric function ϵ of LSCO at $p = 0.24$. These data are obtained from a mix of two techniques, namely the infrared reflectivity between 2.5 meV and 0.5 eV and ellipsometry from 0.5 to 5 eV. The part 0.5–5 eV is using a fit to the ellipsometry in order to extrapolate the reflectivity data at higher photon energy and extract ϵ through Kramers–Kronig relations.

C. STUDY OF THE PLANCKIAN MODEL ($\nu = 1$)

Here, we study the Planckian model defined by Eqs. (7) and (10) of the main text. We calculate the quasiparticle mass, derive approximate analytical expressions for the self-energy and the frequency-dependent optical conductivity, and check these approximations against the numerically computed conductivity. We finally discuss the ω/T scaling properties of the model.

1. Single-particle self-energy

We implement the ultraviolet cutoff in Eq. (7) by limiting the imaginary part of the self-energy to the constant value $\mathrm{Im}\,\Sigma(\Lambda) = -g\pi k_B T S(\Lambda/k_B T)$ for all energies $|\varepsilon| > \Lambda$. The real part contains a constant term $\mathrm{Re}\,\Sigma(0)$ that is compensated by a shift of chemical potential and can therefore be subtracted from $\Sigma(\varepsilon)$. The remaining real part describes the renormalization of the single-particle dispersion by inelastic scattering processes, which at leading order amounts to a renormalization of the mass. The renormalized single-particle mass or quasiparticle mass is

$$\frac{m^*_{\mathrm{qp}}}{m} = 1 - \frac{d}{d\varepsilon}\mathrm{Re}\left[\Sigma(\varepsilon) - \Sigma(0)\right]_{\varepsilon=0} \equiv \frac{1}{Z}, \tag{S8}$$

where Z is the quasiparticle residue. After evaluating the derivative of $\Sigma(\varepsilon)$ using Eq. (7), performing an integration by parts, and separating the even and odd terms in the resulting integral, we arrive at the expression:

$$\begin{aligned}\frac{m^*_{\mathrm{qp}}}{m} &= 1 + g\int_0^{\frac{\Lambda}{k_B T}} dx\,\frac{S'(x) - S'(-x)}{x}\\ &= 1 + 2g\int_0^{\frac{\Lambda}{k_B T}} dx\,\frac{\sinh x - x}{x(\cosh x - 1)}.\end{aligned} \tag{S9}$$

The function to integrate approaches $1/x$ at large x and the integral therefore approaches $\ln(\Lambda/k_B T) + \mathrm{cste}$ if $\Lambda \gg k_B T$. We write this as

$$\frac{m^*_{\mathrm{qp}}}{m} = 1 + 2g\ln\left(a\frac{\Lambda}{k_B T}\right) \qquad (\Lambda \gg k_B T), \tag{S10}$$

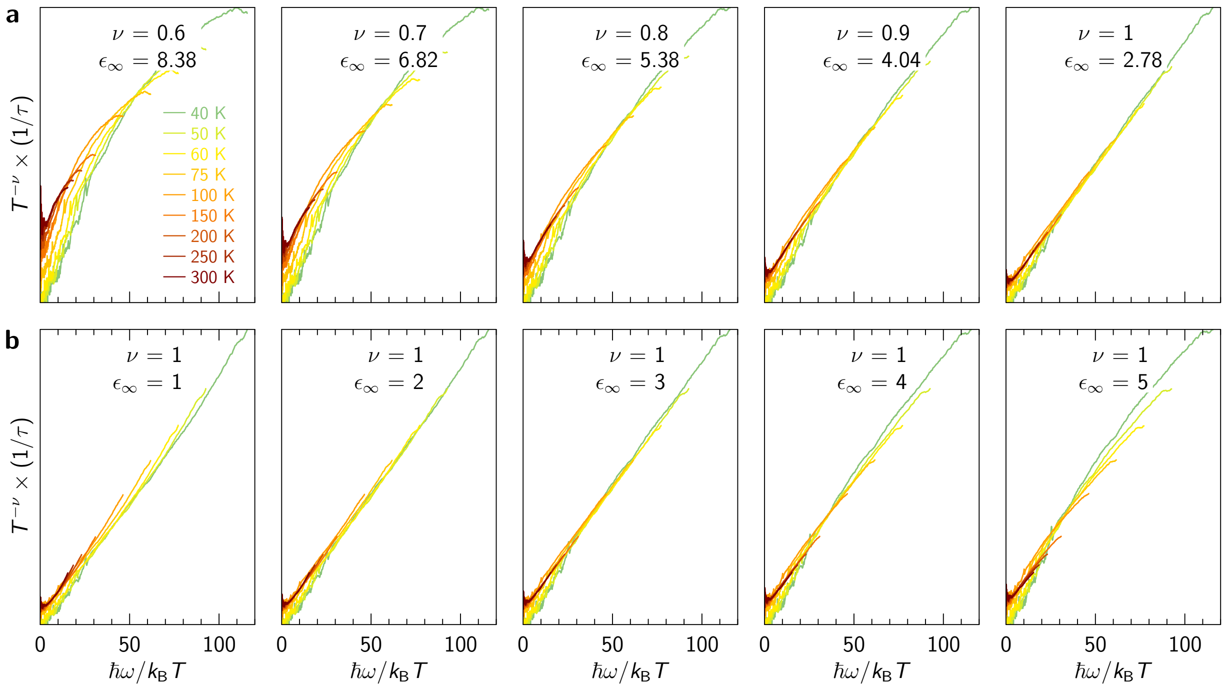

Supplementary Fig. 2. Evidence of Planckian scaling for the scattering rate. a Optimal ω/T scaling of the scattering rate for the values of ν indicated in each panel; the value of ϵ_∞ is the one ensuring the best collapse of the curves. **b** Sensitivity of the scaling collapse to the value of ϵ_∞ for $\nu = 1$. The vertical axis starts at zero in each panel and is otherwise arbitrary, unless a value of the spectral weight K is specified.

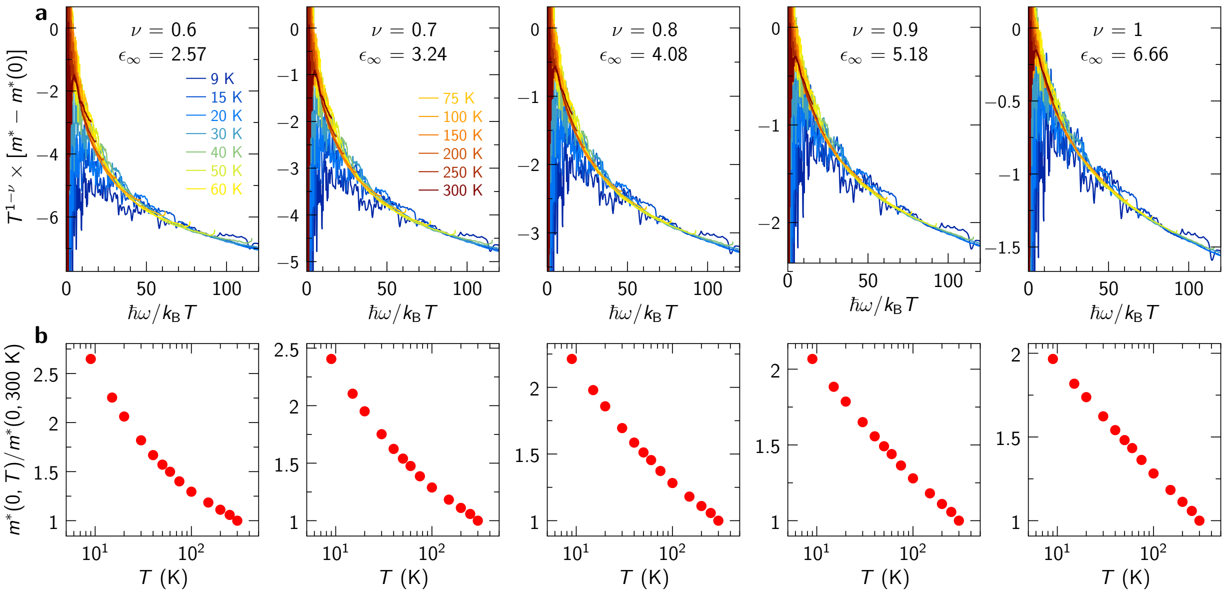

Supplementary Fig. 3. Evidence of Planckian scaling for the mass enhancement. a Optimal ω/T scaling of the mass enhancement for the values of ν indicated in each panel; the value of ϵ_∞ is the one ensuring the best collapse of the curves. **b** Values of $m^*(0)$ that give the best collapse.

where the constant a is given by

$$a = \lim_{x_c \to \infty} \frac{1}{x_c} \exp\left[\int_0^{x_c} dx \, \frac{\sinh x - x}{x(\cosh x - 1)}\right] = 0.770542.$$

If $\varepsilon \gg k_B T$, the integral giving $\Sigma(\varepsilon)$ is dominated by the region $|x| \gg 1$ and we can replace the scaling function by its asymptotic form $S(|x| \gg 1) = |x|$. We then find

$$\mathrm{Re}\left[\Sigma(\varepsilon) - \Sigma(0)\right] \approx g\varepsilon \ln\left|\frac{\varepsilon^2}{\Lambda^2 - \varepsilon^2}\right| + g\Lambda \ln\left|\frac{\Lambda - \varepsilon}{\Lambda + \varepsilon}\right| \quad \text{(S11)}$$

in this regime, which will be used below in building approximations for the conductivity.

2. Optical conductivity

Supplementary Fig. 4 shows the optical conductivity calculated for weak coupling ($g = 0.04$) and strong coupling ($g = 4$) at a temperature $k_BT = 10^{-3}\Lambda$, and plotted using the cutoff Λ as the unit of energy. One can distinguish three regimes of frequency, as pointed out in Ref. 2.

In the regime (I), the frequency is lower than the temperature. The scattering rate approaches a frequency-independent value proportional to T and the conductivity approaches a Drude form with renormalized spectral weight $Z\Phi(0)$. To obtain the asymptotic expression of the conductivity, we expand numerator and denominator in Eq. (10) around $\omega = 0$ to get

$$\sigma_{(\mathrm{I})}(\omega) = i\hbar\Phi(0)\int_{-\infty}^{\infty} d\varepsilon\, \frac{-df(\varepsilon)/d\varepsilon}{\hbar\omega\left[1 - d\Sigma(\varepsilon)/d\varepsilon\right] - 2i\mathrm{Im}\,\Sigma(\varepsilon)}.$$

At low enough temperature, the derivative of the Fermi function is sharply peaked at $\varepsilon = 0$ and the denominator in the function to integrate can be approximated by its value at $\varepsilon = 0$. Since the imaginary part of the self-energy is an even function of ε, the term in square brackets becomes $1 - d\mathrm{Re}\,\Sigma(\varepsilon)/d\varepsilon|_{\varepsilon=0} = 1/Z$. Furthermore, Eq. (7) gives $\mathrm{Im}\,\Sigma(0) = -2\pi g k_BT$, leading to

$$\sigma_{(\mathrm{I})}(\omega) \approx \frac{Z\Phi(0)}{-i\omega + 4\pi g Z k_BT/\hbar}. \qquad \text{(S12)}$$

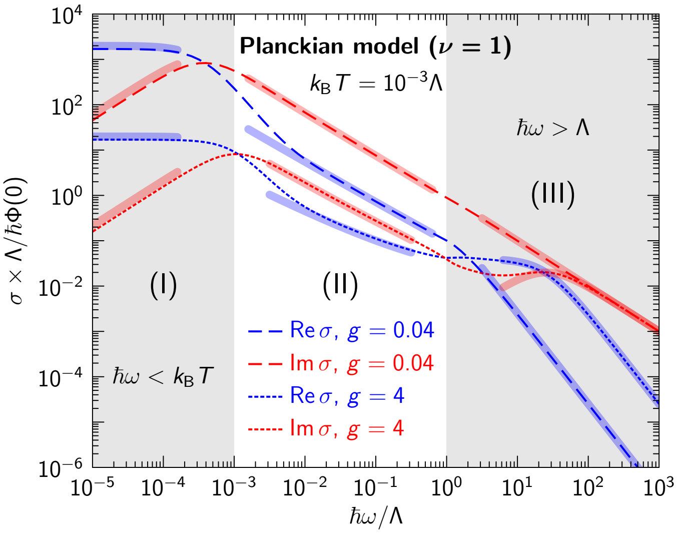

Supplementary Fig. 4. **Optical conductivity of a Planckian model.** Real (blue) and imaginary (red) parts of the optical conductivity, Eqs. (10) and (7) of the main text, for $g = 0.04$ (dashed) and $g = 4$ (dotted) at temperature $k_BT = 10^{-3}\Lambda$. The thick lines show Eqs. (S12), (S15), and (S14) in the corresponding regime of frequency.

This has the usual dissipative ($\mathrm{Re}\,\sigma > \mathrm{Im}\,\sigma$) Drude structure with a scattering time $\tau = \hbar/(4\pi g k_BT)$, corresponding to a T-linear resistivity. The coefficient A of the resistivity deduced from Eq. (S12) is smaller than the exact result, Eq. (13) of the main text, by a factor $7\zeta(3)/\pi^2 = 0.853$. The predictions of Eq. (S12) with $1/Z$ given by Eq. (S10) are displayed in Supplementary Fig. 4 as the thick lines in the regime (I).

In the regimes (II) and (III), the frequency is large compared with temperature and we can therefore replace the Fermi functions by their expression at $T = 0$:

$$\begin{aligned}\sigma(\hbar\omega \gg k_BT) &= \frac{i\Phi(0)}{\omega}\int_{-\hbar\omega}^{0}\frac{d\varepsilon}{\hbar\omega + \Sigma^*(\varepsilon) - \Sigma(\varepsilon + \hbar\omega)}\\ &\approx \frac{i\hbar\Phi(0)}{\hbar\omega - 2\Sigma(\hbar\omega/2)}.\end{aligned} \qquad \text{(S13)}$$

At the second line, we have approximated the function to integrate by its value in the middle of the integration window, noting that the real (imaginary) part of $\Sigma(\varepsilon)$ is odd (even) in ε — provided that the constant $\mathrm{Re}\,\Sigma(0)$ is subtracted from $\Sigma(\varepsilon)$, which is implicit in Eq. (S13). The condition $\hbar\omega \gg k_BT$ allows us to use Eq. (S11) and the asymptotic form $S(x) = |x|$ when evaluating $\mathrm{Im}\left[-2\Sigma(\hbar\omega/2)\right] = \pi g \min(\hbar\omega, 2\Lambda)$. In the regime (III), the real part of $\Sigma(\hbar\omega/2)$ disappears from the conductivity because the right-hand side of Eq. (S11) drops as Λ/ε, such that we arrive at the following approximation:

$$\sigma_{(\mathrm{III})}(\omega) \approx \frac{\Phi(0)}{-i\omega + \pi g \min(\omega, 2\Lambda/\hbar)}. \qquad \text{(S14)}$$

This is asymptotically an inductive regime ($\mathrm{Im}\,\sigma > \mathrm{Re}\,\sigma$), where the real part decreases as $1/\omega^2$ and the imaginary part as $1/\omega$, such that $|\sigma_{(\mathrm{III})}| \sim 1/\omega$. As the asymptotic conductivity is purely imaginary, $\arg(\sigma_{(\mathrm{III})})$ approaches the value $\pi/2$. This is illustrated in Supplementary Fig. 5. Equation (S14) agrees with the numerics, as the thick lines show in the regime (III) of Supplementary Fig. 4.

For the regime (II), we expand Eq. (S11) for $\hbar\omega \ll \Lambda$ and arrive at the expression

$$\sigma_{(\mathrm{II})}(\omega) \approx \frac{\Phi(0)}{-i\omega}\,\frac{1}{1 + 2g\left[1 - \ln\left(\frac{\hbar\omega}{2\Lambda}\right)\right] + i\pi g}, \qquad \text{(S15)}$$

which is also in good agreement with the numerics (thick lines in the regime (II) of Supplementary Fig. 4). Without the cutoff-dependent logarithmic correction, $\sigma_{(\mathrm{II})}(\omega)$ would display pure $\sigma \propto (-i\omega)^{-1}$ behavior, as may be expected in a quantum critical system with linear-in-energy single-particle scattering rate [2]. This is realized for $g \to 0$, as shown by the dashed lines in Supplementary Fig. 4. For finite g, however, the logarithmic correction reduces the decay rate of both real and imaginary parts (dotted lines).

We see in Supplementary Fig. 4 that the approximation Eq. (S15) captures the change of behavior of the conductivity with increasing g in the intermediate frequency regime $k_BT < \hbar\omega < \Lambda$. We therefore use this approximation in

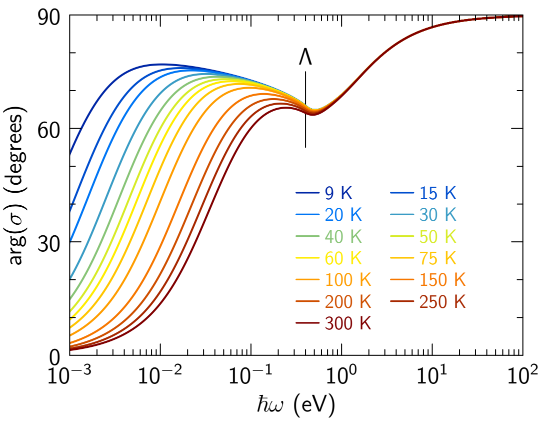

Supplementary Fig. 5. Full frequency dependence of arg(σ). Crossover from the regime of effective exponent $\nu^* < 1$ for $\hbar\omega < \Lambda$ to the asymptotic regime showing $\nu = 1$ and $\arg(\sigma) = \pi/2$ for $\hbar\omega > \Lambda$. The model parameters are $g = 0.23$ and $\Lambda = 0.4$ eV.

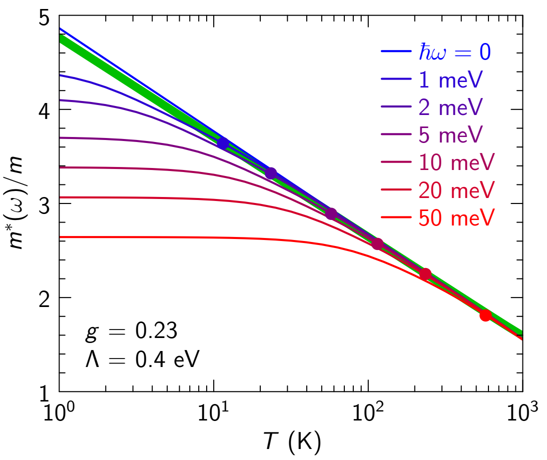

Supplementary Fig. 6. Optical mass versus quasiparticle mass. Temperature-dependent optical mass enhancement predicted by Eqs. (10) and (7) of the main text for $g = 0.23$, $\Lambda = 0.4$ eV and various frequencies, compared with the quasiparticle mass from Eq. (S10) in green. The dots indicate $\hbar\omega = k_B T$. For $k_B T > \hbar\omega$, we have $m^*(\omega) \approx m^*_{qp}$.

order to extract the apparent power-law exponent of $|\sigma|$ by computing the logarithmic derivative at $\hbar\omega = \Lambda/2$, near the middle of domain (II). We thus arrive at Eq. (12) of the main text.

3. ω/T scaling

As the approximations presented so far take one of the two limits $\omega \ll T$ or $\omega \gg T$, they cannot predict the form of the ω/T scaling expected when ω and T are comparable. If the self-energy were obeying the scaling property $\Sigma(\varepsilon) = T\mathscr{F}(\varepsilon/T)$, then also the conductivity, Eq. (10), would obviously scale exactly like $1/\sigma(\omega) = TF(\omega/T)$. The cutoff that must be introduced in Eq. (7) breaks this property, since the self-energy is rather of the form $\Sigma(\varepsilon) = T\mathscr{F}(\varepsilon/T, \Lambda/T)$. The perfect scaling of $1/\sigma$ is therefore lost as well. In the following, we obtain closed formula for the approximate scaling of $1/\sigma$.

At low energy and temperature, $\mathrm{Im}\,\Sigma$ is not influenced by the cutoff, such that the ideal scaling $1/\sigma \propto -i\omega m^*/m + 1/\tau = TF(\omega/T)$ is expected to hold reasonably well for the real part, i.e., $1/\tau \approx Tf_\tau(\omega/T)$. This expectation is confirmed by the numerical simulations (Fig. 5**a** of the main text). Equation (S12) indicates that the function $f_\tau(x)$ is close to $4\pi g$ at $x = 0$, which is confirmed as well by the numerics. Since this corresponds to $-2\mathrm{Im}\,\Sigma(0)/k_B T$, it is tempting to infer that $1/\tau$ is close to twice the single-particle scattering rate, which would give $2\pi g S(x)$ for the scaling function. However, that function goes to $2\pi g x$ at large x, while the simulations go to $\pi g x$. We therefore try $f_\tau(x) = 2\pi g S(x/2)$, which works well despite small deviations at low x, as seen in Fig. 5**a** of the main text. This result could have been directly guessed from Eq. (S13).

Since Eq. (S13) correctly predicts the approximate scaling of $1/\tau$, we use this same relation for m^*, which leads to $m^*/m \approx 1 - (2/\hbar\omega)\mathrm{Re}[\Sigma(\hbar\omega/2) - \Sigma(0)]$. This expression immediately gives $m^*(0)/m \approx 1 - \Sigma'(0) = m^*_{qp}/m$, a result confirmed by the numerics (see Supplementary Fig. 6). If the function $S(x)$ is replaced by the very similar but simpler function $|x| + 2e^{-|x|/2}$, which has the same behavior at low and high values of x, a complete evaluation of $\mathrm{Re}[\Sigma(\varepsilon) - \Sigma(0)]$ becomes possible. We thus find that $m^*(\omega) - m^*(0)$ scales as $f_m(\omega/T)$ with

$$f_m(x) = 2g\left\{1 - \gamma - \ln\left(\frac{x}{4}\right) + \frac{2}{x}\left[e^{\frac{x}{4}}\mathrm{Ei}\left(-\frac{x}{4}\right) - e^{-\frac{x}{4}}\mathrm{Ei}\left(\frac{x}{4}\right)\right]\right\}. \tag{S16}$$

$\gamma = 0.577$ is Euler's constant and Ei is the exponential integral function. The function f_m is compared in Fig. 5**b** of the main text with the numerical simulations. The slight difference is a consequence of using the approximation Eq. (S13) for computing the optical mass, not a consequence of approximating the function $S(x)$.

To conclude this section, we note that the presence of ω/T scaling has been previously tested in Bi2212 by considering the quantity $\hbar/(k_B T \mathrm{Re}\,\sigma)$ [2]. Equation (14) of the main text shows that $\mathrm{Re}\,\sigma$ is independent of ϵ_∞ and K, making it the ideal quantity for testing the presence of ω/T scaling in the raw data. In our model, however, this quantity does not scale with ω/T, as seen in Supplementary Fig. 7. Using the form $\sigma \propto 1/(-i\omega m^*/m + 1/\tau)$ and our observations that, to a very good accuracy, $\hbar/\tau = k_B T f_\tau(\omega/T)$ and $m^*/m = m^*_{qp}/m + f_m(\omega/T)$, we deduce

$$\frac{\hbar}{k_B T \mathrm{Re}\,\sigma} \sim f_\tau\left(\frac{\omega}{T}\right) + \frac{(\hbar\omega/k_B T)^2}{f_\tau(\omega/T)}\left[\frac{m^*_{qp}}{m} + f_m\left(\frac{\omega}{T}\right)\right]^2.$$

Due to the logarithmic variation of m^*_{qp} with temperature, the second term on the right-hand side is not a function of ω/T. Despite the conspicuous absence of scaling, the behavior of the model in a narrow range of ω/T (Supplementary Fig. 7**b**) and for temperatures above 100 K is strikingly sim-

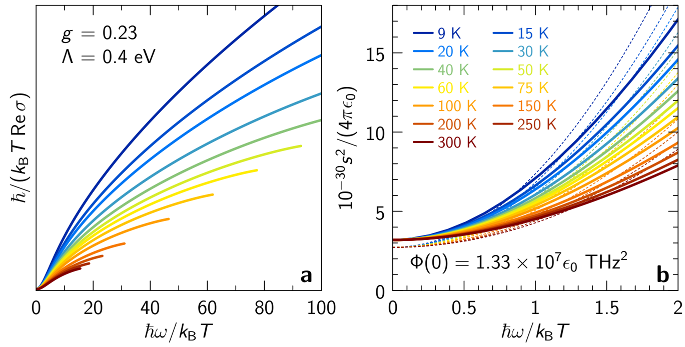

Supplementary Fig. 7. Scaling violation in Re σ. Absence of ω/T scaling in $\hbar/(k_BT\,\mathrm{Re}\,\sigma)$ evaluated numerically in the Planckian model over **a** a wide and **b** a narrow range of ω/T. The dotted lines in **b** show the approximation Eq. (S17).

ilar to that reported in Ref. 2, suggesting that the model could be useful to analyze Bi2212 data as well. In particular, $\hbar/(k_BT\,\mathrm{Re}\,\sigma)$ increases as $(\omega/T)^2$ as pointed out in Ref. 2, albeit with a T-dependent curvature. Since this curvature is in principle accessible in the raw optical data, it is interesting to relate it to the parameters of our Planckian model. Using our approximate scaling functions, we find for $\omega\to 0$:

$$\frac{\hbar}{k_BT\mathrm{Re}\,\sigma}\approx\frac{4\pi g}{\Phi(0)}\left\{1+\left[\frac{1}{48}+\left(\frac{m^*_{\mathrm{qp}}/m}{4\pi g}\right)^2\right]\left(\frac{\hbar\omega}{k_BT}\right)^2\right\}. \tag{S17}$$

This expression is in reasonable agreement with the exact numerical result (see dotted lines in Supplementary Fig. 7**b**). Equation (S17) can in principle be used to extract the three parameters of the theory all at once from the raw optical data. This endeavor is somewhat risky, though, because the approximate validity of Eq. (S17) is limited to a very narrow low-frequency range, where optical data is usually noisy. Furthermore, the sensitivity to the cutoff Λ is only logarithmic.

D. STUDY OF THE SUB-PLANCKIAN MODEL ($\nu<1$)

Here, we introduce and study a model with sub-linear energy and temperature dependencies of the self-energy. We follow the same outline as for the Planckian model, first discussing the self-energy, then the optical conductivity, and finally the ω/T scaling. Beside presenting the model, our main goal is to argue that it gives predictions that are in disagreement with the experimental observations in LSCO at doping p^*, *including* for the apparent exponent $\nu^*<1$ of the optical conductivity.

1. Single-particle self-energy

The central property of the class of theories discussed in this paper is that the single-particle scattering rate is local (independent of momentum) and assumes the form $-\mathrm{Im}\,\Sigma(\varepsilon)=\pi g(k_BT)^\nu S_\nu(\varepsilon/k_BT)$, where $S_\nu(0)$ is finite and $S_\nu(|x|\gg 1)\propto|x|^\nu$. These two conditions ensure that the dc resistivity behaves as T^ν and the ac scattering rate approaches ω^ν for $\hbar\omega>k_BT$, while at intermediate frequencies the scattering rate divided by T^ν is a function of ω/T. The case $\nu=1$ is the Planckian model described in Supplementary Information Sec. C, while here we consider the case $\nu<1$. An important aspect of the sub-Planckian model is that it does not require an ultraviolet cutoff, because the Kramers–Kronig integral giving the real part of the self-energy is now convergent. Thus the theory is completely specified in terms of the low-energy properties of the carriers, which then determine the thermodynamic, transport, and spectroscopic properties. This is an important difference relative to the case $\nu=1$, where the high-energy structure of the theory, represented by the ultraviolet cutoff, controls the thermodynamics and the optical mass.

Certain microscopic models with conformal invariance realize exactly the scaling form of the single-particle scattering rate with $\nu<1$ and provide an explicit expression for the function $S_\nu(x)$ [3–6]. We borrow our self-energy model from those:

$$\Sigma(z)=g(k_BT)^\nu\int_{-\infty}^{\infty}dx\,\frac{S_\nu(x)}{z/k_BT-x} \tag{S18a}$$

$$S_\nu(x)=\frac{(2\pi)^\nu}{\pi\Gamma(1+\nu)}\cosh(x/2)\left|\Gamma\left(\frac{1+\nu}{2}+i\frac{x}{2\pi}\right)\right|^2, \tag{S18b}$$

where Γ denotes the Euler gamma function. Note that g is dimensionfull with the unit of energy to the power $1-\nu$. Proceeding like in the case $\nu=1$, we find that the quasiparticle mass diverges at low temperature like $T^{\nu-1}$:

$$\frac{m^*_{\mathrm{qp}}}{m}=1+g(k_BT)^{\nu-1}c_\nu,\quad c_\nu=\int_0^\infty dx\,\frac{S'_\nu(x)-S'_\nu(-x)}{x}.$$

This is a robust property of the sub-Planckian model — it does not depend on the particular scaling function $S_\nu(x)$ — that disagrees with the logarithmic temperature dependence observed experimentally in LSCO at doping $p=0.24$. Arguably, for ν close to unity it is difficult to distinguish the power law $T^{\nu-1}$ from a logarithmic temperature dependence.

In order to derive approximations for the conductivity, it is helpful to estimate the self-energy at energies larger than k_BT. For $\varepsilon\gg k_BT$, we replace $S_\nu(x)$ by its asymptotic value, which is $|x|^\nu/\Gamma(1+\nu)$, and we obtain for the real part:

$$\mathrm{Re}[\Sigma(\varepsilon)-\Sigma(0)]=-g\frac{\pi\tan(\pi\nu/2)}{\Gamma(1+\nu)}\mathrm{sign}(\varepsilon)|\varepsilon|^\nu\quad(\varepsilon\gg k_BT). \tag{S19}$$

2. Optical conductivity and ω/T scaling

The frequency-dependent conductivity crosses over from the dissipative Drude regime to the asymptotic inductive

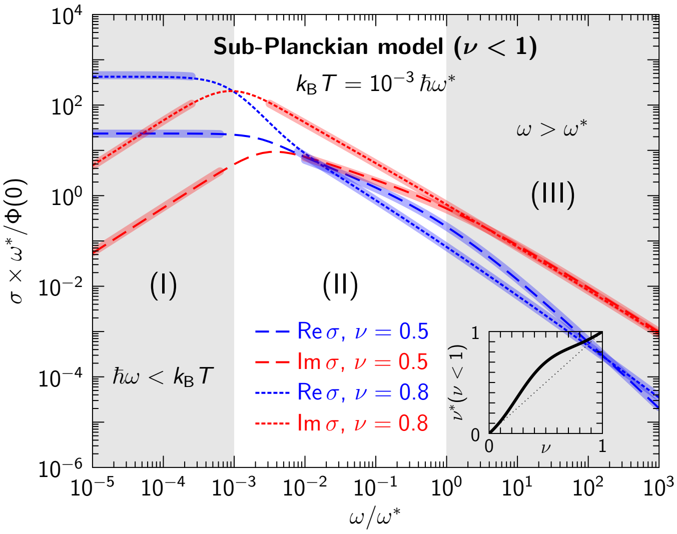

Supplementary Fig. 8. Optical conductivity of a sub-Planckian model. Real (blue) and imaginary (red) parts of the optical conductivity, Eqs. (10) of the main text and (S18), for $\nu = 0.5$ (dashed) and $\nu = 0.8$ (dotted) at temperature $k_B T = 10^{-3}\,\hbar\omega^*$. The thick lines show Eqs. (S21) and (S22). Inset: effective exponent as given by Eq. (S23).

regime via an intermediate regime whose extension is controlled by g and ν (while for $\nu = 1$, it is controlled by the cutoff Λ). We define a crossover frequency ω^* by the condition $\hbar\omega^* = 2\mathrm{Re}[\Sigma(\hbar\omega^*) - \Sigma(0)]$: for $\omega < \omega^*$, the self-energy dominates in Eq. (10) while for $\omega > \omega^*$, one approaches the regime where $\mathrm{Re}\,\Sigma$ becomes irrelevant. Using Eq. (S19), we find

$$\hbar\omega^* = \left[\frac{2\pi g \tan(\pi\nu/2)}{\Gamma(1+\nu)}\right]^{\frac{1}{1-\nu}}. \qquad \text{(S20)}$$

If the temperature and the frequency are both measured in units of ω^*, the conductivity multiplied by ω^* becomes a function of ω/ω^* and T/ω^* that depends on ν, but no longer on g. This function is displayed in Supplementary Fig. 8 for two values of ν. The change of behavior around ω^* is clearly visible.

In the Drude regime, we proceed like for $\nu = 1$ and get

$$\sigma_{(\mathrm{I})}(\omega) \approx \frac{Z\Phi(0)}{-i\omega + 2gZ\frac{[\Gamma(\frac{1+\nu}{2})]^2}{\Gamma(1+\nu)}(2\pi k_B T)^\nu/\hbar}, \qquad \text{(S21)}$$

as indicated by the thick lines in the regime (I) of Supplementary Fig. 8. This shows that the resistivity is proportional to T^ν, another robust property of the sub-Planckian model that disagrees with the linear resistivity observed in LSCO at $p = 0.24$.

In the regimes (II) and (III), we use the approximation Eq. (S13), together with the asymptotic self-energy given by Eq. (S19) for the real part and by $-\pi g|\varepsilon|^\nu/\Gamma(1+\nu)$ for the imaginary part, yielding

$$\sigma_{(\mathrm{II,III})}(\omega) \approx \frac{\Phi(0)}{-i\omega + 2^{1-\nu}\pi\hbar^{\nu-1}g\frac{1-i\tan(\pi\nu/2)}{\Gamma(1+\nu)}\omega^\nu}. \qquad \text{(S22)}$$

This expression reproduces the behavior proportional to i/ω required by causality in the limit $\omega \to \infty$, irrespective of the value of ν, and interpolates well across ω^*, as shown by the thick lines in Supplementary Fig. 8. In the regime (II), a nontrivial power law emerges with an apparent exponent $\nu^* > \nu$. The logarithmic derivative of $|\sigma_{(\mathrm{II,III})}|$ evaluated at $\omega^*/2$ gives the exponent

$$\nu^*(\nu < 1) = \nu + \frac{\left(2^{2\nu-1} + 2^{4\nu-2}\right)(1-\nu)}{2^{2\nu} + 2^{4\nu-2} + 1/\sin^2(\pi\nu/2)}. \qquad \text{(S23)}$$

We emphasize that this formula is not valid for $\nu = 1$. The cases $\nu = 1$ and $\nu < 1$ have different analytic structures, with the consequence that the apparent exponent in Eq. (S23) is independent of the coupling g, while in the Planckian case, Eq. (12) of the main text, it depends only on g. The function (S23) is displayed as an inset in Supplementary Fig. 8. An effective exponent of 0.8, as observed experimentally in LSCO, requires $\nu = 0.61$. This in turn implies a resistivity $\rho \sim T^{0.61}$, in marked disagreement with experiments, so that a sub-Planckian interpretation is not tenable.

Supplementary Fig. 9**a** and Supplementary Fig. 9**b** show the modulus and argument of the optical conductivity calculated numerically using Eqs. (10) and (S18) with $\nu = 0.61$. The coupling constant g is fixed such that the magnitude of the conductivity is similar to that in Fig. 3 of the main text. This calculation reproduces the power law observed experimentally in the modulus, but predicts a behavior of the argument that agrees less well with experiment than the model with $\nu = 1$, especially at low frequency (compare

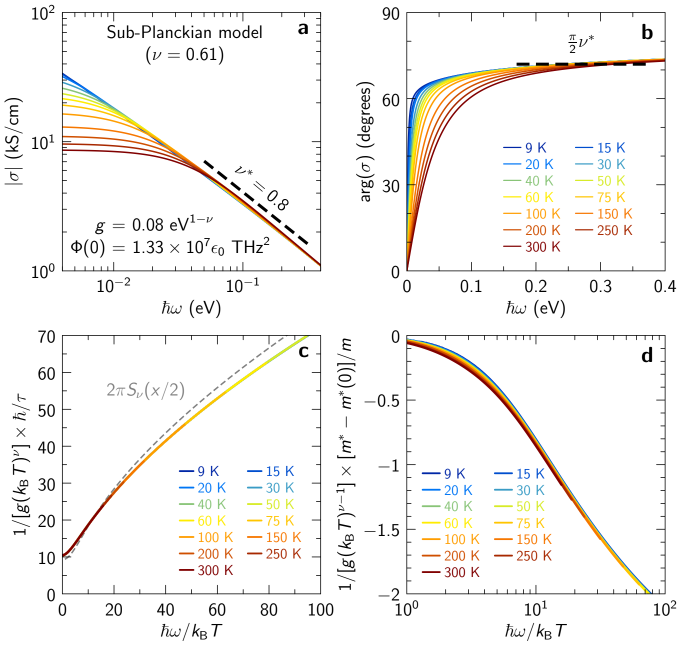

Supplementary Fig. 9. Effective exponent and scaling for the sub-Planckian model. **a** Modulus and **b** phase of the optical conductivity given by Eqs. (10) of the main text and (S18) with the parameters indicated in **a**. **c** Approximate collapse of the scattering rate and **d** mass enhancement.

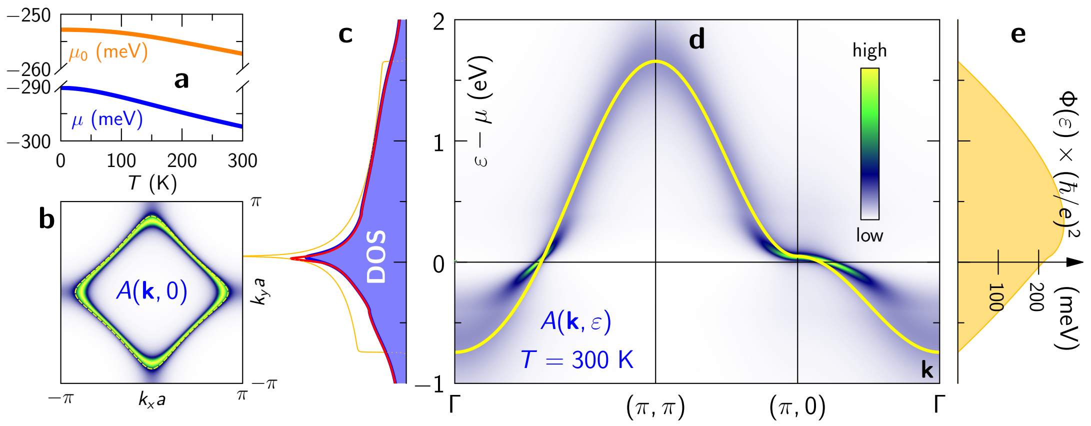

Supplementary Fig. 10. **Tight-binding model for LSCO at $p = 0.24$. a** Temperature dependence of the noninteracting (orange) and interacting (blue) chemical potential. **b** Noninteracting Fermi surface (dotted) and zero-energy spectral function at $T = 300$ K (same color scale as in **d**). **c** Noninteracting (orange) and interacting density of states at $T = 10$ K (red) and $T = 300$ K (blue). **d** Tight-binding band (yellow) and spectral function at $T = 300$ K. **e** Transport function. The energies in **c**, **d**, and **e** are measured relative to the corresponding chemical potential.

Figs. 3**b**, 3**d**, and Supplementary Fig. 9**b**). Supplementary Fig. 9**c** and Supplementary Fig. 9**d** show that $1/\tau$ and m^*, after being properly scaled (and divided by g to produce dimensionless quantities), display a good collapse as a function of ω/T. This justifies the scaling laws employed in Supplementary Information Sec. B for $\nu < 1$. The most striking disagreement with experiment is seen in the scattering rate. Indeed, the scaling requires to normalize $1/\tau$ by T^ν, which does not lead to a good collapse of the experimental data for $\nu \approx 0.6$ (see Supplementary Fig. 2).

E. TIGHT-BINDING MODEL AND ONE-PARTICLE PROPERTIES

Here, we present the tight-binding model that is used in the main text for fixing the values of the band mass m and spectral weight K. We discuss the properties of the model and show how these properties are modified by the self-energy.

The one-particle properties of the model are displayed in Supplementary Fig. 10. The tight-binding dispersion with up to second-neighbor hopping amplitudes is given by $\varepsilon_\mathbf{k} = -2t[\cos(k_x a) + \cos(k_y a)] - 4t' \cos(k_x a)\cos(k_y a)$ with $t = 0.3$ eV, $t'/t = -0.17$, and $a = 3.78$ Å. All properties are shown for an electron density $n = 0.76/a^2$, i.e., a hole doping $p = 0.24$. The noninteracting chemical potential μ_0 varies from -253 meV at $T = 0$ to -257 meV at $T = 300$ K (Supplementary Fig. 10**a**). The corresponding tight-binding band with energies measured relative to $\mu_0(T = 0)$ is shown in Supplementary Fig. 10**d**, and the associated density of states in Supplementary Fig. 10**c**. The Fermi-level DOS is 1.646 $\text{eV}^{-1}a^{-2}$, which corresponds to a band mass $m = 2.76 m_e$. The Fermi surface is closed around the Brillouin-zone center, as seen in Supplementary Fig. 10**b**.

The transport function

$$\Phi(\varepsilon) = 2e^2 \int_\mathrm{BZ} \frac{d^2k}{(2\pi)^2} \left(\frac{1}{\hbar}\frac{d\varepsilon_\mathbf{k}}{dk_x}\right)^2 \delta(\varepsilon + \mu_0 - \varepsilon_\mathbf{k}) \qquad \text{(S24)}$$

is displayed in Supplementary Fig. 10**e**; the $T = 0$ value of $(\hbar/e)^2\Phi(0)$ is 211 meV.

The self-energy, Eq. (7) of the main text, renormalizes the dispersion and consequently all one-particle properties. The chemical potential calculated for $g = 0.23$ and $\Lambda = 0.4$ eV is shown in Supplementary Fig. 10**a** and varies from -290 meV at $T = 0$ to -297 meV at $T = 300$ K. We emphasize that the high-energy details of the self-energy influence the value of μ, such that this model calculation, although internally consistent, is not expected to be realistic for LSCO as it misses those high-energy non-universal aspects. The downward renormalization of the chemical potential by the interaction implies a renormalization of the Fermi surface (maximum of the zero-energy spectral function), as seen in Supplementary Fig. 10**b**. A change of the Fermi-surface volume at fixed density seems to violate Luttinger's theorem. The theorem is not applicable here, however, because it requires a true Fermi surface — a discontinuity of the momentum distribution. Since the self-energy does not vanish on the Fermi surface, there is no such discontinuity in the model. The color map in Supplementary Fig. 10**d** shows the renormalization of the tight-binding band, which, since the self-energy is local, is the same at all wave-vectors. The corresponding interacting DOS is plotted in Supplementary Fig. 10**c** for two temperatures.

F. EFFECT OF PARTICLE-HOLE ASYMMETRY

In the main text, we compare the optical spectra of LSCO with microscopic models that possess particle-hole (p-h) symmetry. The cuprate materials break p-h symmetry at the

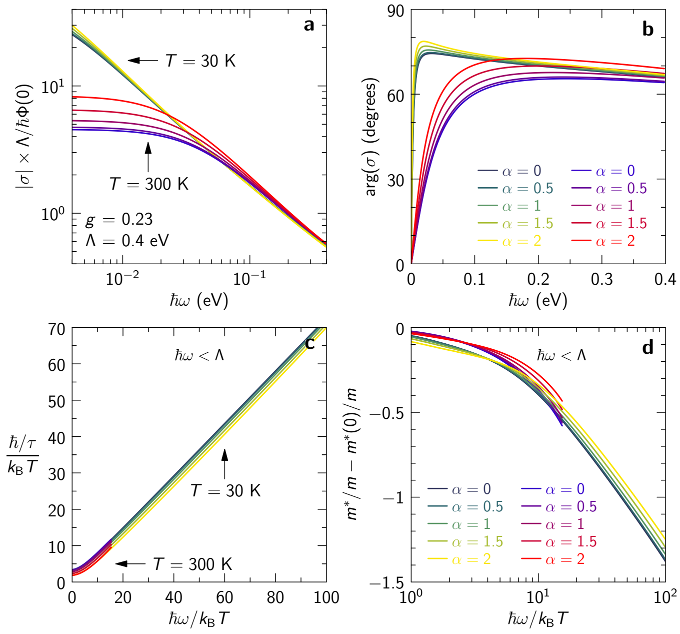

Supplementary Fig. 11. **Weak effect of particle-hole asymmetry.** **a** Modulus and **b** phase of the optical conductivity given by Eqs. (10) and (S25) with $\nu = 1$ and the other parameters as indicated in **a**; the dependence on α is shown for low and room temperature in blue–yellow and blue–red shades, respectively. **c** Corresponding α dependence of the scattering rate and **d** the mass enhancement.

level of the band structure and, consequently, also at the level of the self-energy. The band-structure effects enter the optical spectra via the energy-dependent transport function $\Phi(\varepsilon)$ replacing $\Phi(0)$ in Eq. (10) of the main text. The transport function is smooth (see Supplementary Fig. 10**e**), unlike the DOS that varies rapidly due to the van Hove singularity (Supplementary Fig. 10**c**). As $\Phi(\varepsilon)$ is almost featureless, we do not expect a significant effect of the band structure on the frequency dependence of the optical spectra. These spectra represent transitions from occupied to empty states and thus mix scattering effects at positive and negative energies. For that reason, they should also not be particularly sensitive to the p-h asymmetry of the self-energy. It is indeed clear that the self-energy is to a large extent averaged between positive and negative energies in Eq. (10). In order to check this expectation and justify our use of p-h symmetric models for data analysis, we consider here a more general model including p-h asymmetry in the self-energy. This model was proposed recently to investigate the thermopower of non-Fermi liquids [7], which is sensitive to p-h asymmetry because, unlike the optical spectra, it vanishes identically for p-h symmetric systems.

Particle-hole asymmetry is introduced in the self-energy by replacing $S_\nu(x)$ in Eq. (S18) by the function [7]

$$S_\nu^\alpha(x) = \frac{(2\pi)^\nu}{\pi\,\Gamma(1+\nu)}\,\frac{\cosh(x/2)}{\cosh(\alpha/2)}\left|\Gamma\left(\frac{1+\nu}{2}+i\frac{x+\alpha}{2\pi}\right)\right|^2. \tag{S25}$$

A positive value of the dimensionless parameter α skews the scattering by enhancing the scattering rate for holes relative to that for particles. In Supplementary Fig. 11, we illustrate the effect of α on the optical conductivity. Results are shown for the Planckian case $\nu = 1$. Similar results are found for $\nu < 1$. Supplementary Fig. 11**a** and Supplementary Fig. 11**b** show that upon increasing p-h asymmetry from zero to $\alpha = 2$, the apparent exponent of the conductivity hardly changes, both at low and room temperatures. Note that $\alpha = 2$ represents a strong p-h symmetry violation: with this value, the scattering rate is ~ 7 times larger for holes at $\varepsilon \ll -k_\mathrm{B}T$ than for electrons at $\varepsilon \gg k_\mathrm{B}T$. Supplementary Fig. 11**c** and 11**d** show that the ω/T scaling laws of the optical scattering rate and mass enhancement also only change in a minor quantitative way upon increasing p-h asymmetry.

REFERENCES

[1] C. Berthod, J. Mravlje, X. Deng, R. Žitko, D. van der Marel, and A. Georges, Non-Drude universal scaling laws for the optical response of local Fermi liquids, Phys. Rev. B **87**, 115109 (2013).

[2] D. van der Marel, H. J. A. Molegraaf, J. Zaanen, Z. Nussinov, F. Carbone, A. Damascelli, H. Eisaki, M. Greven, P. H. Kes, and M. Li, Quantum critical behaviour in a high-T_c superconductor, Nature **425**, 271 (2003).

[3] I. Affleck and A. W. W. Ludwig, Critical theory of overscreened Kondo fixed points, Nucl. Phys. B **360**, 641 (1991).

[4] D. L. Cox and A. Zawadowski, Exotic Kondo effects in metals: Magnetic ions in a crystalline electric field and tunnelling centres, Adv. Phys. **47**, 599 (1998).

[5] O. Parcollet, A. Georges, G. Kotliar, and A. Sengupta, Overscreened multichannel SU(N) Kondo model: Large-N solution and conformal field theory, Phys. Rev. B **58**, 3794 (1998).

[6] P. T. Dumitrescu, N. Wentzell, A. Georges, and O. Parcollet, Planckian metal at a doping-induced quantum critical point, Phys. Rev. B **105**, L180404 (2022).

[7] A. Georges and J. Mravlje, Skewed non-Fermi liquids and the Seebeck effect, Phys. Rev. Research **3**, 043132 (2021).
